# Supplementary material for: Focal Loss Analysis of Nerve Fiber Layer Reflectance for Glaucoma Diagnosis
Source: Transl Vis Sci Technol. 2021 May 6;10(6):9. doi: 10.1167/tvst.10.6.9 (PMC8107497; doi:10.1167/tvst.10.6.9)

# Supplementary

Our azimuthal spatial frequency filtering method is based on the assumption that incidence angle variation along the circumpapillary circles has first-degree sinusoid azimuthal variation. The phase and magnitude of this variation depends on the geometry of the eye, as well as the position of the OCT scan beam relative to the pupil. Generally, the incidence angle is positive (centripetal) because the scan-mirror conjugate plane in the pupillary plane is anterior to the center of curvature of the retina (i.e., the retina appears concavely curved on an OCT cross-section). When the OCT beam is centered in the pupil, the retina nasal to the disc has the largest off-perpendicular incidence angle while the retina temporal to the disc has near-perpendicular incidence. This corresponds to a first-degree sinusoidal variation in the azimuthal dimension when the reflectance map is transformed into a polar coordinate centered on the disc. Because the centration of the OCT beam, axial eye length, and retinal curvature varies between eyes and scans, the amplitude and phase of this azimuthal variation also varies, but it is always primarily a first-degree sinusoidal variation. Therefore a band-stop filter that remove the first-degree sinusoid component in the azimuthal dimension suppresses the reflectance measurement bias caused by incidence-angle variation.

We designed an experiment to confirm the assumption. The incident angle and reflectance along the circle with D=3.4mm were calculated from 35 normal eyes. The incident angle of each sample points was estimated based on the nearby NFL plane (Fig. S1A). Along the 3.4-mm diameter circle, the incident angle indeed showed the expected first-degree azimuthal sinusoidal variation with amplitudes ranging from 1.5° to 9.6° with a constant offset of 2.8 to 11.1° (Fig. S1B). Due to the centripetal offset, the absolute value of the incidence angle also generally varied as a first-degree sinusoid along the analytic circle around the disc, with an amplitude of 6.1±3.7°, significantly above zero (p<0.001).

The NFL reflectance decreased when the absolute incidence angle (zero angle defined as perpendicular incidence) increased (Fig. S1C). Furthermore, the amplitude of first-degree azimuthal sinusoidal reflectance variation was significantly correlated with the first-degree sinusoidal variation in absolute incidence angle variation (r=0.421, p=0.007). In contrast, the amplitudes of other azimuthal sinusoidal orders, i.e., 0, 2, 3, 4, were not significantly correlated (p>0.17). In summary, reducing the first-degree azimuthal sinusoidal reflectance variation could reduce the bias caused by incident angle.

There were also second-degree sinusoidal variations in incidence angle (Fig. S1B) associated with normally thicker NFL locations superiorly and inferiorly. Our azimuthal filtering preserved this spatial frequency component as it contained diagnostic information.

To implement the azimuthal filter, we constructed a band-stop filter along the azimuthal direction. First, the reflectance map was transformed from Cartesian coordinate to polar coordinate, with the disc center as origin. Then the reflectance map was transformed to the Fourier domain. As it was in polar coordinate, the horizontal direction of the spectrum corresponds to the azimuthal direction, while the vertical direction corresponds to the radial direction. A band-stop filter was set to block only the first-degree angular frequency component in azimuthal direction. A low-pass filter was also performed on both azimuthal and radial direction to reduce speckle noise. After the filtering, the map was transformed back to the space domain.

We compared the diagnostic accuracy of NFL reflectance parameters obtained with and without azimuthal filtering and found that the accuracy of all parameters was always better with filtering on.


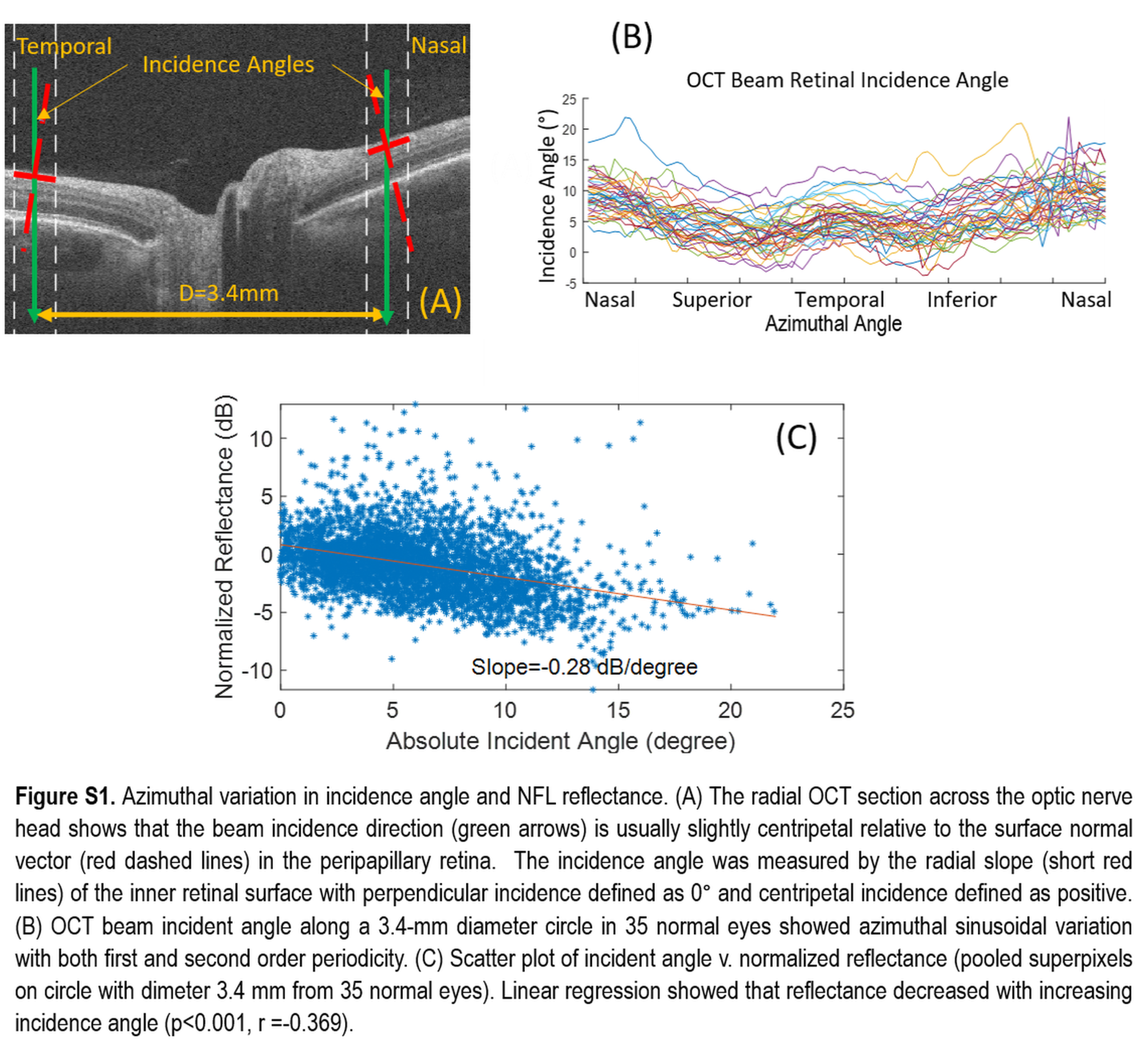

Supplement: Supplement 1 [file tvst-10-6-9_s001.docx]
